# Teaching Improvisation through Processes. Applications in Music Education and Implications for General Education

**DOI:** 10.3389/fpsyg.2017.00911

**Published:** 2017-06-02

**Authors:** Michele Biasutti

**Affiliations:** FISPPA Department, University of PadovaPadova, Italy

**Keywords:** musical improvisation, music creativity, cognitive processes, cognitive models, pedagogy on processes

## Abstract

Improvisation is an articulated multidimensional activity based on an extemporaneous creative performance. Practicing improvisation, participants expand sophisticated skills such as sensory and perceptual encoding, memory storage and recall, motor control, and performance monitoring. Improvisation abilities have been developed following several methodologies mainly with a product-oriented perspective. A model framed under the socio-cultural theory of learning for designing didactic activities on processes instead of outcomes is presented in the current paper. The challenge is to overcome the mere instructional dimension of some practices of teaching improvisation by designing activities that stimulate self-regulated learning strategies in the students. In the article the present thesis is declined in three ways, concerning the following three possible areas of application: (1) high-level musical learning, (2) musical pedagogy with children, (3) general pedagogy. The applications in the music field focusing mainly on an expert's use of improvisation are discussed. The last section considers how these ideas should transcend music studies, presenting the benefits and the implications of improvisation activities for general learning. Moreover, the application of music education to the following cognitive processes are discussed: anticipation, use of repertoire, emotive communication, feedback and flow. These characteristics could be used to outline a pedagogical method for teaching music improvisation based on the development of reflection, reasoning, and meta-cognition.

## Introduction

Music improvisation could be defined as the real-time creative performance of novel music and consists of inventing music extemporaneously. Improvisation is a discovery act that goes beyond the application of a preexisting technique and aims to delineate an original framework. Improvisation could be considered one of the most articulated expressions of creative behavior as stated by Beaty (2015, p. 109): “The improvising musician faces the unique challenge of managing several simultaneous processes in real-time—generating and evaluating melodic and rhythmic sequences, coordinating performance with other musicians in an ensemble, and executing elaborate fine-motor movements—all with the overall goal of creating esthetically appealing music.” Improvisation is a multidimensional articulated action that requires diverse refined abilities. Improvisation involves both the skills of singing or playing a musical instrument, and generating new music. Instrumental techniques and creative processes are simultaneously activated to create a musical piece. The real-time nature of the performance induces a situation in which the performers are under the pressure of promptly creating the music. The improvisation behaviors activate information processing, which has to be coordinated and internalized to obtain a fluid performance. While the singer or the instrumentalist are performing, low-level cognitive and motor processes are automatized and the cognitive resources can focus on higher-order processes.

Improvisation also has a versatile and interactive nature: a performance can be modeled and adapted to different circumstances. The adjustments involve feedback processes which consist of real-time answers to situational events: the musical improvisations are modified and adjusted in relation to the conditions of the performance. The cognitive components are developed in a social context: improvisation is an interpersonal action that is developed among people cooperating to create a musical piece extemporaneously (Monson, 1996; MacDonald et al., 2011; Kenny, 2014; Schober and Spiro, 2014; Morgan et al., 2015; Wilson and MacDonald, 2017). The social aspect is a core feature of improvisation which involves musical and collective norms that have to be respected (Berliner, 1994). Performers develop complex non-verbal communication skills for working together proficiently and collaborating is the crucial characteristic of a musical improvisation (Sawyer, 2011).

Improvisation can also be a teaching technique to innovate the current methods bringing new challenges to education (Santi, 2016). Improvisation can open new perspectives regarding teaching/learning, proposing a model based on creativity development and interactive knowledge construction. Luquet (2015) argued that everything he knows about teaching he learned from jazz. In the society of knowledge characterized by the instant availability of information there has been a change in the paradigm of teaching and learning:

“Whereas at one time teaching and learning was information being passed, memorized, and repeated, students can now find their own knowledge. Learning now consists of using information in creative ways and requires a shift in how students are taught. This is quite similar to what occurred in music over a hundred years ago when jazz was introduced to the culture. Music moved from musicians playing what the composer wrote, to creating their own music within a structure. This shift is an apt metaphor for the changes being felt in twenty-first century teaching and learning. The processes of learning and creating jazz provide a way to illustrate new teaching methods that allow students to discover new knowledge through their own creative interests and to develop self-efficacy with the material.” (Luquet, 2015, p. 60).

For these reasons jazz (and improvisation) could be considered as a model of teaching. The current article aims to propose an educational approach for the development of processes rather than products (Monk, 2012; Biasutti, 2015), focusing on an expert's use of improvisation. This approach is framed under the socio-cultural theory of learning (Vygotsky, 1978; Wegerif, 2008) which defines an active role for the student. The main focus of the article is on teaching music improvisation. Moreover, a more generalized learning context is given in the final section which proposes implications of the framework to broader learning.

## Educational benefits of improvisation

The educational benefits that improvisational activities could have on the students is an increasingly discussed issue in the field of music education. Aspects such as historical significance, the position of improvisation in music education, stimulated musical abilities and the activation of transfer processes were considered (Azzara, 2002; Sarath, 2002; Parncutt, 2006; Hickey, 2009; Sawyer, 2011).

Improvisation has been an essential component of musical expression and several forms have been used throughout history. The analysis of improvisation techniques occurring at different times shows the relevance of historical and stylistic variables. In the nineteenth century, improvisation was abandoned with the use of scores. Until this period, improvisation was used for educating several complex musical abilities (Parncutt, 2006). Historical and stylistic elements are relevant since they provide a reference for practicing improvisation. However, improvisation is often relegated to a peripheral position in current music educational programs, which focus primarily on the improvement of performance and convergent abilities.

Several researchers call for a more relevant role of improvisation in music education (Azzara, 2002; Campbell, 2009; Hickey, 2009; Beegle, 2010; Wright and Kanellopoulos, 2010; Sawyer, 2011; Burnard, 2012). Improvisation offers the possibility to link informal and formal learning and to acquire a holistic music education by merging ear training, music theory and performance in a learning environment full of stimuli (Campbell, 2009). Recently, improvisation was introduced in progressive music academies in the framework of programs such as early music, organ, jazz, ethnomusicology, and music education. Improvisation could be used to educate students at any level, but the possibility to attend music improvisation classes has to be available to all instrumental pupils (Parncutt, 2006). Improvisation classes could challenge students in developing a more mindful approach to learning than traditional didactic techniques by using a practice focused on reflection and creative production.

Several authors believe that improvisation is one of the most formative musical practices stimulating pupils' melodic, harmonic, rhythmic and expressive sensibility. In addition, there is a complete involvement of the performer in the promotion of a cohesive musical piece using perceptual, compositional and performance skills. During improvisation higher-order abilities are educated (Azzara, 2002; Azzara and Snell, 2016), inducing a superior comprehension of the relationship between music performed with and without sheet music. Reading a score could become a passive action when the student considers only the written notes and does not dedicate the proper attention to musical communication with the other performers and the audience. Improvisation provides stimuli to the music player to reflect on the qualities of the sound produced developing a real-time sound dialog with the other performers. These aspects could induce a deeper awareness in the players even when they are reading a score.

There is evidence that the development of improvisational skills largely depends on the interaction with the environment and on variables such as motivation, self-efficacy and constancy in exercising rather than being completely innate. Improvisation practice activates several cognitive and social processes (Sawyer, 2011), and learning to improvise could be particularly beneficial for students at all levels (Parncutt, 2006). Several pieces of research highlight that introducing improvisation at the very beginning of musical education would be highly advantageous for the students. Improvisation activates several cognitive processes and enhances the coordination of complex abilities. In addition, improvisation is also used as a rehabilitation technique on executive functions in older participants (Biasutti and Mangiacotti, 2017) and in psychiatry (Degli Stefani and Biasutti, 2016).

Improvisation induces processes of transfer of learning and contributes to the improvement of closed abilities in other areas, such as language (Berkowitz, 2010). Both music and language have common aspects such as a grammar and a set of conventions, and improvisation practice could play a relevant part in developing abilities for speech (Harrison and Pound, 1996). Improvisation activities during childhood offer the possibility to experience essential activities of expression through music. Learning jazz has been considered a means for improving general skills such as idea generation, decision making, and synthesis of ideas. Moreover, improvisation involves collaboration and the development of social skills (Sawyer, 2011) that are traditionally difficult to teach and develop.

## Techniques for teaching improvisation

Several techniques have been used for teaching and learning music improvisation in genres such as jazz. Informal learning was the most relevant aspect of learning jazz in the past and trainee musicians' attendance to performances was a relevant activity. When jazz programs were established in music academies this tradition was abandoned introducing formal courses and precise curricula based on instruction. One of the most well-known methods is supported on theory and on the exercising of scales, arpeggios, chords, and harmonic progressions (Huovinen et al., 2011). The acquisition of the grammar of musical improvisation was the main activity whereas the jazz culture, the importance of playing by ear and other informal learning activities were considered less important or secondary. Aural imitation skill is one of the crucial abilities supporting jazz improvisation achievement (Palmer, 2016). Most of the improvisation pedagogical approaches focus on basic skills development and improvisation teaching often involves isolated melodic, harmonic and rhythmic exercises. Jazz standards are commonly considered some of the most important activities for practicing improvisation. One technique is the “riff” approach, which includes learning jazz models which are played over different chord progressions (Hickey, 2009). Other techniques regard activities such as practicing with minus one recording, transcribing solos by ear, sight reading, and arranging music pieces. “In addition to theoretical knowledge and scale and chord practice, more innovative proposals include activities such as aural skill development, playing by ear and providing an authentic learning environment in which opportunities to play with more experienced improvisers are offered” (Biasutti, 2015, p. 6).

An approach based on awareness of skills development rather than instruction could be beneficial to the students. The tradition of free expression in improvisation could be considered by merging expressive and technical elements to enhance the comprehension of music as a language (Borgo, 2007). Kratus (1991, 1996) proposes a model of improvisation pedagogy linking intuitive with expert and musically refined behaviors. A seven-level sequential model has been theorized which includes a succession of progressively complex actions from exploration to personal improvisation. The advantages of this model are that the activities are independent of age and the musical features are in relation to the students' level of knowledge and skills. In this way it is possible to start teaching improvisation at an elementary level. In addition, improvisation is considered along a continuum with a direct connection between the various levels.

## A process-oriented teaching of music improvisation

The literature review provides few educational applications of the cognitive research on music improvisation. A process-oriented teaching method could be beneficial in music improvisation at several levels including students in music academies. A teaching technique for the enhancement of processes rather than products can provide inputs for developing specific skills such as problem solving and critical thinking to assist a reflective practice during improvisation (Biasutti, 2013).

**Figure 1:**
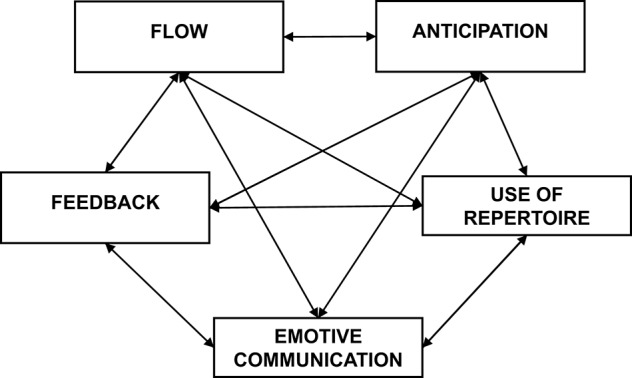
The dimensions of musical improvisation from Biasutti (2015).

A reflective method that overcomes the mere instructional level of some current practices of teaching improvisation in jazz pedagogy is aimed for. In a pedagogical approach on processes, the teacher focuses on the development of the necessary actions for improvising. The actions are then connected in a comprehensive scenario to maintain the development of improvisation. In a process-oriented approach the teacher is a facilitator for supporting the development of students' ideas. A method of improvisation based on cognitive processes could be developed considering the five dimensions proposed by Biasutti and Frezza (2009): anticipation, use of repertoire, emotive communication, feedback and flow (see Figure 1) which are described in the following paragraphs.

### Anticipation

**Anticipation** involves thinking in advance about elements at the melodic, harmonic and rhythmic levels during musical improvisation (Pressing, 1988; Kenny and Gellrich, 2002). Anticipation contributes to idea generation and to the decision-making process. Anticipation facilitates finding complex solutions in the improviser and involves deciding the sound events that can be used in a musical context instantaneously (De Dreu et al., 2012). In anticipating the sound events, the performer develops planning abilities and the idea of the total structure of the improvisation.

KEY CONCEPT 1 **Anticipation**

Thinking in advance of elements at the melodic, harmonic and rhythmic levels during musical improvisation.

Several educational activities could be used for developing anticipation skills, such as designing an improvisation in detail. The generation of ideas has to follow a context and a strategy for the development of these actions enhances the anticipation process. The musical practice could propose tasks such as thinking aloud about the development of an improvisation and describing it. Another task could involve the performance of an improvisation with an instrument then stopping to play the instrument to continue with solo singing.

### Use of repertoire

The **use of repertoire** regards the application of melodic and rhythmic figures such as licks (short musical motifs) during musical improvisation. This repertoire of licks is stored in the long-term memory and is learned through listening and studying the improvisations of eminent musicians. The use of the repertoire is based on memorization of patterns that could be used during improvisation and involve several levels. The aim is to expand both the long-term memory archive of patterns and licks and the schemes for managing them during improvisation. The challenge is to provide students not only the basic material (the licks), but also the strategies for properly selecting the licks (the grammar). The selection of licks has to be framed respecting the characteristics of the context of the improvisation. For this reason a proper method of education must provide strategies not only for the memorization of licks, but also for the correct use and for the re-elaboration and adaptation of licks to a specific situation.

KEY CONCEPT 2 **Use of repertoire**

Utilizing repertoire formulas such as clichés or licks during improvisation.

Several activities could be proposed for expanding the students' repertoire of licks such as listening and analysis of solos of famous musicians in different musical styles. The educational approach consists of learning by ear and the aim is to stimulate the students to repeat and memorize the solo, then to sing the solo and finally to play the solo with an instrument. The practice could involve the analysis of previous solos, the identification of relevant parts and of the strategies used for developing the solo. A reflective thinking approach has to be promoted in the students, stimulating their reasoning on how to make the licks meaningful in specific situations. In addition, creativity principles also have to be introduced, by asking students to create variations and their personal adaptations of licks.

### Emotive communication

**Emotive communication** regards the induction and transmission of affective and emotional feelings during musical improvisation. The reference for musical communication is the musical grammar and the stylistic principles, which are used as a set of rules for developing the piece. During emotive communication the melodic, harmonic, timbre, dynamic and rhythmic elements are modulated and idea generation is mediated by emotions and inner feelings (Jordanous and Keller, 2012). The more knowledge the performer has about the musical grammar the more advanced are the musical emotions that s/he can convey. In addition, there is a circular process: “when semantics—the meaning of music, for the student—is regarded as more important than syntax (the melodic, rhythmic and harmonic musical structures), syntactic patterns become paradoxically easier to learn (Parncutt, 2006, p. 92).

KEY CONCEPT 3 **Emotive communication**

Communicating emotions through improvisation.

Several educational activities could be used for developing emotive communication such as inviting pupils to improvise on a specific emotive state. In this activity students have to find the proper sound for expressing their inner feelings activating a process of introspection. In addition, a verbalization task of the experience could be included, in which students are asked to reflect on the sound produced and whether the sounds matched their inner feelings. This verbalization activity leads students to reflect upon the process of sound selection and production and to define the variables of emotive communication in music. The objective is to stimulate self-reflection and an approach to improvisation based on reasoning rendering the implicit process of emotive communication a conscious one. Activities on emotive communication imply switching one's focus from the formal aspects to the dynamic and expressiveness of the music providing students with a more motivating and significant field of work than traditional approaches.

### Feedback

**Feedback** regards the skills to reply to events by producing real-time adaptations to advance an improvisation. These modifications are made for rendering the improvisation more reliable and logical. Feedback involves different monitoring processes according to the features of the music, the group dynamics and the context. Several strategies for managing feedback are used by performers, and several kinds of feedback have been described, such as external and internal feedback (Biasutti and Frezza, 2009), and short-term and long-term feedback (Pressing, 1988). External feedback is the communication between players, the environment and the audience while internal feedback is when the performer regulates his/her behavior by focusing on the perceptions about his/her performance (Biasutti and Frezza, 2009). Short-term feedback is the operation to control definite motor actions, while long-term feedback contributes to choosing the proper actions and in making decisions (Pressing, 1988).

KEY CONCEPT 4 **Feedback**

Making real-time changes to synchronize or improve an improvisation using monitoring processes.

The educational activities on feedback aim to increase students' skills to respond promptly to the external inputs during an improvisation. While improvising, several unexpected events could happen and performers must be educated on how to manage these issues. Feedback activities could involve tasks such as proposing musical events which change suddenly, requesting participants to react to these modifications while performing. Another activity could involve a dialog with musical questions and answers, focusing on aspects of music such as melody, harmony, timbre, and rhythm. These actions could be performed even with eyes closed to enhance a deeper concentration on elements of the sound events. Additional feedback activities could propose events from other perceptive modalities such as visual and kinesthetic stimuli, asking students to provide a musical interpretation of these stimuli. To enhance interaction among performers risk-taking tasks (Wopereis et al., 2013) could also be considered in which performers interact challenging each other in generating original ideas and variations.

### Flow

**Flow** is an intense state of consciousness including physiological, cognitive and affective aspects during musical improvisation. Flow is linked to optimal experience (Csikszentmihalyi, 1990; Chirico et al., 2015; Croom, 2015; Páez et al., 2015; Habe and Tement, 2016), a state in which performers give their best. Performers experiencing flow have constructive feelings and perceive the activity as enjoyable (Csikszentmihalyi and Rich, 1997). Flow can enhance improvisation inducing a sense of spontaneity and a natural flow of musical ideas. Flow is a comprehensive multidimensional construct and the following nine aspects are embodied: concentration on the task, sense of control, clear goals, challenge and skill balance, the merging of action and awareness, unambiguous feedback, a deformed sense of time, loss of self-consciousness, and an autotelic experience (Biasutti, 2011).

KEY CONCEPT 5 **Flow**

A state of mind of intense absorption during improvisation, including cognitive, affective and physiological aspects, linked to optimal experience.

Teaching flow directly is demanding and educational activities are based on promoting a performance setting that facilitates flow. Several actions could be considered for these purposes so as to set-up clear objectives that can be verified in a direct way. The activities could be based on the concept of zone of proximal development considering the distance between the current development level and potential level of development that can be achieved by students (Vygotsky, 1978). The level of difficulty has to be matched with the current skills of the performers providing them with appropriate challenges. If the activity becomes boring a more complex level of difficulty has to be proposed. Teachers have to offer positive feedback to the students providing clear suggestions on how to improve the performance. The improvisation activity must provide positive feelings and increase the motivation of the participants. Motivation to learn is an important catalyst to the learning process. Table 1 includes the supporting educational strategies associated with the five improvisation processes.

**Table 1:** Educational techniques in improvisation processes.

**Improvisation process**	**Educational techniques**
Anticipation	- Defining the context and determining the limits of the improvisation. - Describing the conditions and designing the improvisation. - Defining the evolution of the improvisation. - Singing to oneself the melody that one is performing. - Practicing thinking the solo aloud.
Use of repertoire	- Analysis of solos. -Singing, playing and learning the improvisations of different musical styles. - Learning by ear. - Providing examples of strategies for using licks. - Acquisition and application of principles for using licks. - Reflective practice on the strategies used.
Emotive communication	- Improvising based on specific feelings. - Verbalizing the emotions conveyed during improvisation. - Reflective practice on emotive communication.
Feedback	- Musical questions and answers. - Dialogues based on parameters such as rhythm, melody, harmony, and timbre. - Arranging improvisational cues that change unexpectedly and abruptly. - Arranging improvisational cues through verbal directions. - Arranging improvisational cues supported by various sensorial modes (aural, visual, kinesthetic, …). - Arranging stimulating and risk-taking musical activities.
Flow	- Eliciting expectations and designing limpid objectives. - Sharing objectives and goals with group mates. - Designing activities that are within the players' ability to perform. - Designing suitable levels of technical and musical situations joined with existing abilities. - When the action becomes boring, proposing a demanding task. - Designing conditions for enjoyment. - Avoiding breaks. - Evaluating progress by checking whether the objectives have been accomplished. - Giving formative feedback. - Reinforcing motivation.

## Discussion

In the present paper, a pedagogical approach on the development of processes was discussed analyzing the following elements: anticipation, use of repertoire, emotive communication, feedback and flow. These features could be used to outline a pedagogical method based on reasoning, to teach music improvisation stimulating meta-cognition (Luquet, 2015). The aim is to provide a theoretical framework for developing a logical approach to teaching that surpasses a simple instructional level. A program on the processes encourages reflection on the activity and the development of meta-cognitive strategies stimulating pupils to reflect upon the actions and to critically evaluate the task performed (Biasutti, 2013).

The surrounding paradigm is the socio-cultural theory of learning and the student-centered approach as applied in music where pupils are active rather than passive learners elaborating their knowledge in a transformative way (Vygotsky, 1978; Wegerif, 2008). In this context teachers have to support the students by offering them challenging tasks and actively using their feedback. Teachers have to shape their teaching programs to the pupils' needs for supporting the development of their self-regulated learning strategies (Wopereis et al., 2013). Self-regulated learning implies that students independently control their learning through actions such as planning and monitoring. Such kinds of processes are difficult to achieve naturally and the teacher has to effectively stimulate the students to be self-regulated and life-long learners (Biasutti and Concina, 2017).

Regarding curriculum planning, teaching improvisation is a very time-consuming and demanding activity. Teachers who use improvisation as a teaching technique must carefully plan all the variables of the activities instead of improvising their actions. The teachers of improvisation are more prepared, more able to handle the unexpected and the unpredictable, and more willing to take risks than traditional teachers. Teaching improvisation involves designing, monitoring and evaluating (Biasutti, 2013). An initial aspect regards defining a context for improvising, providing a framework to the students and asking them to improvise within determined limits. It is a comfort zone in which students can experiment and discover the extent to which they can make music respecting these limits (Parncutt, 2006).

Designing the activities, teachers should provide a unified plan for process development. In addition, a conscious expression of aesthetic features of the musical productions has to be considered. Teachers have to reinforce the students' intrinsic motivation to music achievement which is directly connected to self-regulated learning. The teaching approach should focus on the learning process rather than on products stimulating pupils' elaboration of musical knowledge in an independent way (Biasutti, 2015). Scaffolding the music student's skills could be intended as one of the most relevant constructive teaching strategies. The learning environment has to be designed to include methods such as learning by doing, inductive learning, critical thinking, and the development of creativity. An interactive communication among teachers and students has to aim at stimulating a consciousness about their role and duties in the students (Santi, 2016). The focus has to be on the quality of the processes rather than on the products for developing the improvisational expertise. A teacher should stimulate students to socialize their implicit knowledge providing them the opportunity to share views and experiences and offering analytical tasks and tools (Luquet, 2015, p. 60).

The current study has limitations. First, there was little space to discuss other improvisation theories and to compare them. Second, the focus was only on the five dimensions of music improvisation according to Biasutti and Frezza (2009) without considering other aspects. The aim was to provide an example that didactic activities are also possible on processes in the music domain and in improvisation. Third, each of the components of Biasutti and Frezza's model are discussed in terms of how each aspect is taught within practice and a further step might be to verify that these strategies do reliably target the intended dimension.

## Further extensions in general education

The theoretical framework of the current essay is based on music with the idea to transfer these principles to other areas (Harrison and Pound, 1996; Berkowitz, 2010). Improvisation is a technique that could be used as an educational model which could be extended in other disciplines. Improvisation could give solutions to questions such as “what does it mean to study today? What are the knowledge construction processes?” Nowadays, a learning model based on memorization, repetition and accumulation is under discussion and considered poor in respect to the complex situations that people face in the everyday life. A learning model based on skills and competency development with actions such as internal elaboration, interaction, connectivity, and shared knowledge building appears more adequate than the traditional one for the current globalized society. Improvisation can innovate education for the creative contribution and the different approach to knowledge building that it conveys (Luquet, 2015, p. 60).

Improvisation could be considered an adaptive behavior to a real-time unpredicted event, based on creativity and divergent skills, providing a wide scale set of stimuli. Improvisation enhances the transfer of learning mechanisms allowing the application of such skills in other domains as well (Harrison and Pound, 1996; Berkowitz, 2010). Fostering improvisational skills would allow the students to develop the abilities to adapt to tomorrow's changing world providing tools for lifelong learning. Furthermore, a way to carry this work forward could be a discussion in terms of its feasibility considering aspects such as how improvisation can be taught by teachers who may not have improvisation skills themselves and how to develop the improvisations skills of teachers.

In conclusion, improvisation is a teaching technique that could give input for renovating current educational methods. Improvisation could open new perspectives regarding teaching and learning, proposing a model based on creativity development and a process oriented teaching approach.

## Author contributions

The author confirms being the sole contributor of this work and approved it for publication.

### Conflict of interest statement

The author declares that the research was conducted in the absence of any commercial or financial relationships that could be construed as a potential conflict of interest.
